# Draft Genome Sequence of *Amphibacillus jilinensis* Y1^T^, a Facultatively Anaerobic, Alkaliphilic and Halotolerant Bacterium

**DOI:** 10.4056/sigs.4107829

**Published:** 2013-07-30

**Authors:** Hong Cheng, Ming-Xu Fang, Xia-Wei Jiang, Min Wu, Xu-Fen Zhu, Gang Zheng, Zhi-Jian Yang

**Affiliations:** 1 College of Life Sciences, Zhejiang University, Hangzhou, P.R.China; 2 Department of Molecular and Cellular Biochemistry, Indiana University, Bloomington, Indiana 47405, United States; 3 State Key Laboratory for Diagnosis and Treatment of Infectious Diseases, First Affiliated Hospital, College of Medicine, Zhejiang University, Hangzhou, China; 4 Ocean Research Center of Zhoushan, Zhejiang University, Zhoushan, P.R.China

**Keywords:** *Amphibacillus*, facultative anaerobe, alkaliphilic bacterium, halotolerant, soda lake, two-component systems

## Abstract

The genus *Amphibacillus* was established in 1990, and seven additional species were described in the past two decades. *Amphibacillus jilinensis* Y1^T^ is a facultatively anaerobic and alkaliphilic bacterium isolated from a soda lake in China. Here we describe the structural and genetic features of the draft genome about the type strain Y1^T^ (3,831,075 bp, with a G+C content of 37.27%). This is the first genome report of the *Amphibacillus* genus.

## Introduction

The genus *Amphibacillus* belongs to the family *Bacillaceae* and was established in 1990 [1]. Currently the genus comprises eight validly published species: *A. xylanus* [1], *A. indicireducens* [2], *A. cookii* [3], *A. marinus* [4], *A. jilinensis* [5], *A. sediminis* [6], *A. fermentum* and *A. tropicus* [7]. All are Gram-positive, moderately alkaliphilic, facultatively anaerobic rods [5,6]. All can grow at pH 9.0 and one can grow at pH 12.0 [2-4,6]. *Amphibacillus jilinensis* Y1^T^ (=CGMCC 1.5123^T^ =JCM 16149^T^) was isolated from a soda lake in Jilin province, China, and grows at pH range from 7.5 to 10.5 with an optimum at 9.0 [5]. Strain Y1^T^ can utilize a large spectrum of substrates as sources of carbon and energy, can grow both aerobically and anaerobically, and tolerate Na^+^ up to 2.8 M. In this genus, three species have been sequenced. A finished genome sequence is *Amphibacillus xylanus* NBRC 15112 (NCBI Accession Number AP012050) and two incomplete sequences are *A. jilinensis* Y1^T^ (NCBI Accession Number AMWI00000000) and *Amphibacillus sediminis* Shu-P-Ggiii25-2 (NCBI BioProject ID PRJDB405) according to the GOLD records [8,9]. Here we report this draft genome of *A. jilinensis* Y1^T^, the first genome from genus *Amphibacillus* to be sequenced.

## Classification and features

A sediment sample was collected from a soda lake (44°45’N, 123°34’E) in Jilin province, China, in November 2007. There is no freshwater river to flow into the lake. Atmospheric water and groundwater are the only water sources of this lake. The lake is rich in Na^+^ (257.2 mg/l), CO_3_^2-^ (50.7 mg/l), Cl^-^ (10.1 mg/l), HCO_3_^-^ (6.5 mg/l) and SO_4_^2-^ (4.4 mg/l), with the pH of the water sample in the same geographical location being 10.0 [5]. The strain Y1^T^ was isolated from enrichment cultures of sediment sample by the Hungate roll-tube technique [10] under a gas phase of O_2_-free N_2_ [1,5].

**Figure 1:**
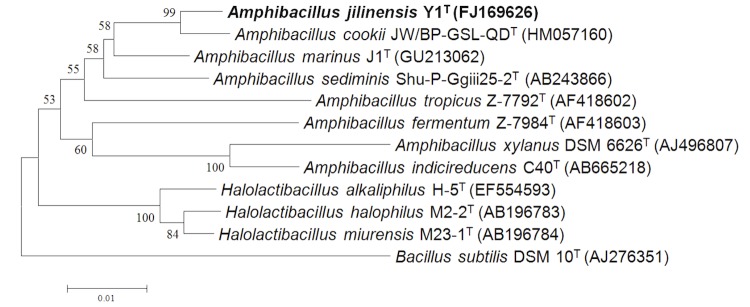
Phylogenetic tree highlighting the position of *A. jilinensis* strain Y1^T^ relative to other type strains within the *Amphibacillus* genus and with the relative *Halolactibacillus* genus. The strains and their corresponding Genbank accession numbers are shown following the organism name and indicated in parentheses. Three strains have their corresponding NCBI genome project IDs and sequencing status [8,13] listed here: PRJNA42371 of *A. xylanus* DSM 6626^T^, complete; PRJNA171498 of ***A. jilinensis* Y1^T^**, Draft; PRJDB405 of *A. sediminis* Shu-P-Ggiii25-2^T^, in progress. The phylogenetic tree uses 16S rRNA gene sequences aligned by the CLUSTALW [14], and phylogenetic inferences were made using Neighbor-joining method based on Tamura-Nei model within the MEGA5 software [15]. Numbers at the branching nodes are percentages of bootstrap values based on 1,000 replications. The scale bar indicates a 1% substitution per nucleotide position. *Bacillus subtilis* DSM 10^T^ was used as an outgroup.

Comparative 16S rRNA gene sequence analysis by BLASTN [11,12] using the NCBI-NR/NT database revealed 93.4-98.8% sequence similarity to members of the genus *Amphibacillus*. Neighbor-Joining phylogenetic analysis based on Tamura-Nei model indicated the taxonomic status of strain Y1^T^ is clearly classified into the same branch with genus *Amphibacillus*, and the most closely related genus is *Halolactibacillus* (Figure 1). *A. jilinensis* Y1^T^ can tolerant high salinity but can also survive without Na^+^. Growth occurs under either aerobic or anaerobic conditions. The optimal growth condition of strain Y1^T^ occurs in medium JY with 0.5 M Na^+^ (0.06 M NaHCO_3_ and 0.44 M NaCl) [5]. The optimum pH is 9.0, with a growth range of pH 7.5-10.5. No growth was observed at pH 7.0 or 11.0. Strain Y1^T^ is mesophilic, with a temperature range of 15-45 ºC and optimum growth at 32 ºC [Table 1]. Cell morphology, motility and sporulation were examined by using transmission electron (H-600, Hitachi) microscopy. Cells of strain Y1^T^ are straight rods with petritrichous flagella, which have a diameter ranging 0.4-0.6 µm and a length of 2.0-3.2 µm (Figure 2a). In the late-exponential and stationary phases of growth, the rods can form terminal endospores (Figure 2b).

**Table 1:** Classification and general features of *A. jilinensis* Y1^T^ according to the MIGS recommendations [16]

**MIGS ID**	**Property**	**Term**	**Evidence code**^a^
		Domain: *Bacteria*	TAS [17]
		Phylum: *Firmicutes*	TAS [18-20]
		Class: *Bacilli*	TAS [21,22]
		Order: *Bacillales*	TAS [23,24]
		Family: *Bacillaceae*	TAS [23,25]
		Genus: *Amphibacillus*	TAS [1,2,6]
	Current classification	Species: *Amphibacillus jilinensis*	
		Type strain: strain Y1^T^ = CGMCC 1.5123 = JCM 16149	TAS [5]
	Gram stain	positive	IDA
	Cell shape	rods	IDA
	Motility	motile	IDA
	Sporulation	sporulating	IDA
	Temperature range	15-45°C	IDA
	Optimum temperature	32°C	IDA
	Carbon source	L-arabinose, cellobiose, D-fructose, D-galactose, D-glucose, lactose, maltose, mannose, D-mannitol, melibiose, D-raffinose, rhamnose, D-sorbitol, sucrose, trehalose and D-xylose.	IDA
	Energy source	yeast extract, sucrose, glucose	IDA
	Terminal electron receptor	Unknown	IDA
MIGS-6	Habitat	aquatic, fresh water, soda lakes, sediment	IDA
MIGS-6.3	Salinity	The water contains Na^+^ (257.2 mg/l), CO_3_^2-^(50.7 mg/l), Cl^-^ (10.1 mg/l), HCO_3_^-^ (6.5 mg/l) and SO_4_^2-^ (4.4 mg/l), with the pH 10.0	IDA
MIGS-22	Oxygen	unknown	IDA
MIGS-15	Biotic relationship	free living	IDA
MIGS-14	Pathogenicity	unknown	
MIGS-4	Geographic location	a soda lake in Jinli Province, P.R. China	IDA
MIGS-5	Sample collection time	November, 2007	IDA
MIGS-4.1	Latitude	44°45’N	IDA
MIGS-4.2	Longitude	123°34’E	IDA
MIGS-4.3	Depth	Sediment	IDA
MIGS-4.4	Altitude	148 m above sea level	IDA

a) Evidence codes - IDA: Inferred from Direct Assay; TAS: Traceable Author Statement (i.e., a direct report exists in the literature); NAS: Non-traceable Author Statement (i.e., not directly observed for the living, isolated sample, but based on a generally accepted property for the species, or anecdotal evidence). These evidence codes are from the Gene Ontology project [26,27]. If the evidence code is IDA, then the property should have been directly observed, for the purpose of this specific publication, for a live isolate by one of the authors, or an expert or reputable institution mentioned in the acknowledgements.

**Figure 2a:**
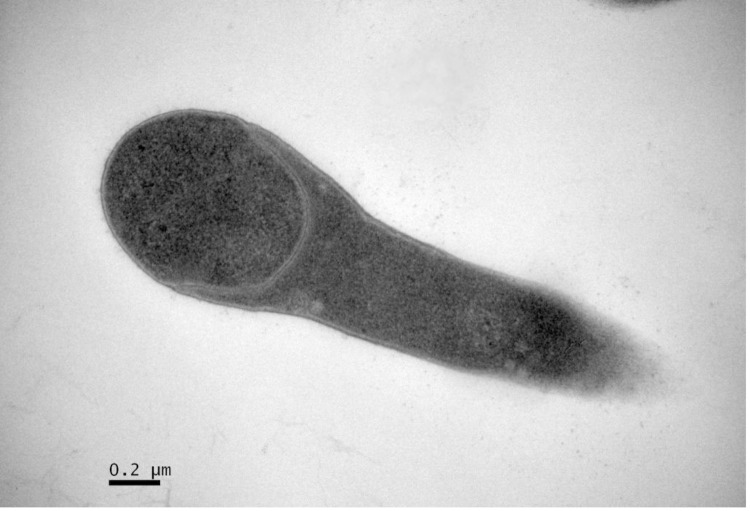
Transmission electron micrograph of cells of strain Y1^T^, showing a longitudinal ultrathin section of a cell forming a spore. Bar: 0.2 μm (a).

**Figure 2b:**
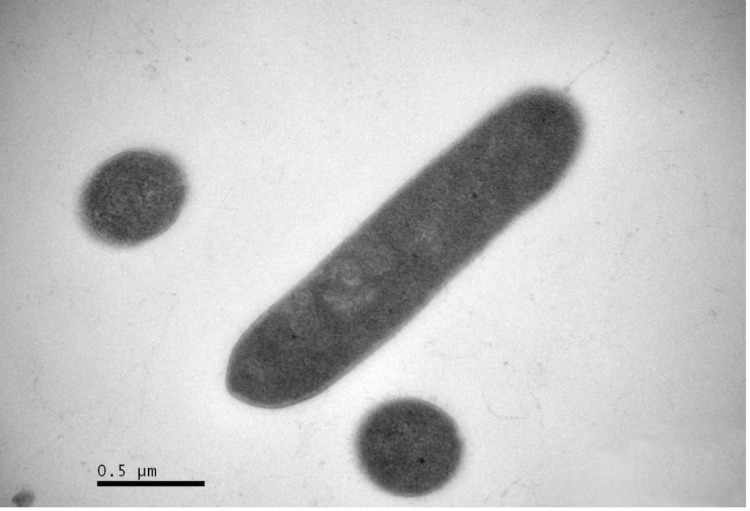
Transmission electron micrograph of cells of strain Y1^T^, showing a longitudinal ultrathin section of the peritrichous flagella in the stationary phase of growth. Bar: 0.5 μm (b).

## Genome sequencing information

### Genome project history

The genome of *A. jilinensis* was selected for next-generation sequencing on the consideration of its facultatively anaerobic characterization and as a new member in genus *Amphibacillus*. This is the first genome report for any of the eight *Amphibacillus* species. Two others are the subject of ongoing own genome projects. This Whole Genome Shotgun project of *A. jilinensis* was deposited at DDBJ/EMBL/GenBank under the accession AMWI00000000 and consists of 83 contigs (further assembling constructed these contigs into 30 scaffolds). Table 2 presents the project information and its association with MIGS version 2.0 compliance [16].

**Table 2:** Project information

**MIGS ID**	**Property**	**Term**
MIGS-31	Finishing quality	High-quality draft
MIGS-28	Libraries used	One pair-end 500 bp library and one pair-end 2 Kb library
MIGS-29	Sequencing platforms	Illumina HiSeq 2000
MIGS-31.2	Fold coverage	130 × (based on 500 bp library), 65 × (based on 2 Kb library)
MIGS-30	Assemblers	SOAP*denovo*
MIGS-32	Gene calling method	RAST
	Genbank ID	AMWI00000000
	Genbank Date of Release	October 18, 2012
	GOLD ID	Gi20767
	Project relevance	Microbial pathway & resources

### Growth conditions and DNA isolation

*A. jilinensis* Y1^T^ was cultivated aerobically in modified JY medium, which contains (per liter distilled water) 2.0 g yeast extract (Difco), 5.0 g sucrose, 0.2 g KCl, 0.2 g KH_2_PO_4_, 0.1 g MgCl_2_. 6H_2_O, 0.5 g NH_4_Cl, 0.1 g CaCl_2_, 0.06 M NaHCO_3_ and 0.44 M NaCl, final pH 9.0 at 32°C for 3 days [5]. Genomic DNA was extracted using the method described by Marmur [28]. The yield, purity and the concentration of genomic DNA was judged by the 0.7% agarose gel electrophoresis with λ-*Hin*d III digest DNA Marker (TaKaRa, Dalian, China) and measured by the NanoDrop 1000 Spectrophotometer (Thermo Fisher Scientific Inc., USA). About 736.6 μg genomic DNA at the concentration 744 ng/μl was obtained.

### Genome sequencing and assembly

Genomic DNA sequencing of *A. jilinensis* Y1^T^ was performed using Solexa paired-end sequencing technology (HiSeq2000 system, Illumina, Inc., USA) [29] with a whole-genome shotgun (WGS) strategy, with a 500 bp-span paired-end library (~500 Mb available reads, ~130-fold genome coverage) and a 2,000 bp-span paired-end library (~250 Mb available reads, ~65-fold genome coverage). All these clean reads were assembled into 83 contigs (the minimum length is 231 bp) and 30 scaffolds (the minimum length is 542 bp) using the SOAP*denovo* v.1.05 [30,31,50]. The quality of the sequencing reads data was estimated by G+C content and sequencing depth correlation analysis.

### Genome annotation

The tRNAs and rRNAs were identified using tRNAscan-SE [32], RNAmmer [33] and Rfam database [34]; The open reading frames (ORFs) and the functional annotation of translated ORFs were predicted and achieved by using the RAST server online [35,51]. Classification of some predicted genes and pathways were analyzed using COGs [36,37] and KEGG [38-40] databases. Meanwhile, we used the InterPro [41,42] to obtain the GO annotation with the database of Pfam [43].

## Genome properties

The draft genome sequence of *A. jilinensis* Y1^T^ revealed a genome size of 3,836,603 bp (scaffold length) and a G+C content of 37.27%. These scaffolds contain 3,649 coding sequences (CDSs), 51 tRNAs (removed 3 Pseudo tRNAs) and incomplete rRNA operons (two 5 S rRNA and one 16 S rRNA). A total of 2,683 protein-coding genes (67.72%) were assigned a predicted function (Table 3) and genes have been categorized into COGs functional groups (Table 4).

**Table 3:** Genome statistics of *A. jilinensis* Y1^T^

**Attribute**	Value	% of total^a^
Genome size (bp)	3,836,603	-
DNA coding region (bp)	3,169,605	82.61
DNA G+C content (bp)	1,429,902	37.27
Total genes^b^	3,705	100.00
RNA genes	56	1.51
Protein-coding genes	3,649	98.49
Genes assigned to COGs	2,683	73.52

a) The total is based on either the size of the genome in base pairs or the total number of protein coding genes in the annotated genome.

b) Includes 1,092 hypothetical proteins and 19 unknown functional proteins by RAST subsystem annotation.

**Table 4:** Number of genes associated with the general COG functional categories

**Code**	**Value**	**%age**^a^	**Description**
J	162	5.37	Translation
K	282	9.34	Transcription
L	201	6.66	Replication, recombination and repair
D	37	1.23	Cell cycle control, mitosis and meiosis
V	88	2.92	Defense mechanisms
T	184	6.10	Signal transduction mechanisms
M	149	4.94	Cell wall/membrane biogenesis
N	72	2.39	Cell motility
U	42	1.39	Intracellular trafficking and secretion
O	90	2.98	Posttranslational modification, protein turnover, chaperones
C	106	3.51	Energy production and conversion
G	359	11.90	Carbohydrate transport and metabolism
E	244	8.08	Amino acid transport and metabolism
F	72	2.39	Nucleotide transport and metabolism
H	79	2.62	Coenzyme transport and metabolism
I	57	1.89	Lipid transport and metabolism
P	159	5.27	Inorganic ion transport and metabolism
Q	36	1.19	Secondary metabolites biosynthesis, transport and catabolism
R	338	11.20	General function prediction only
S	261	8.65	Function unknown
-	966	26.47	Not in COGs

^a^The total is based on the total number of genes which categorized into COGs functional groups in the annotated genome.

## Insights from the genome sequence

The genomic annotation results suggest that strain Y1^T^ can adapt to an extremely basic environments. A large number of genes related to carbohydrate metabolism can encode proteins that provide a stable energy supply to maintain the lower internal pH despite the high external pH [44]. Several cation/proton antiporters were found in the genome, which are also crucial for the maintenance of internal pH [45]. However, the lower number of these genes in Y1^T^ when compared to *Bacillus pseudofirmus* OF4 [44] may imply another way of importing protons into the cell. Meanwhile, as a facultatively anaerobic bacterium, 27 oxidative stress related genes are found in the predicted annotations, such as manganese superoxide dismutase (EC 1.15.1.1), superoxide dismutase [Cu-Zn] precursor (EC 1.15.1.1), organic hydroperoxide resistance transcriptional regulator and CoA-disulfide reductase (EC 1.8.1.14). For facultatively anaerobic strains, these superoxide dismutases (SODs) may be critical because the systems can help to regulate intracellular oxidative stress when the cells grow during aerobic respiration, and can also be used in the treatment of disease, study of pharmacological activity [46] and in the cosmetic industry. It also contains 34 two-component system genes that encode response regulators and sensor histidine kinases. The two-component systems appear to be used to respond to a wide variety of stimuli, including the presence of nutrients, antibiotics and chemoattractants in the environment, changes in osmolarity, temperature, pH, etc [47,48]. This is especially true in strain Y1^T^, in which these systems are thought to be used for recognizing environmental pH, and regulating its internal osmotic stress to survive various environments [49]. According to the database Pfam [43], there are also 9 CRISPRs-associated (Cas) proteins or Cas protein families in this genome of *A. jilinensis*.

## Conclusion

Strain Y1^T^ is the fifth member of the genus *Amphibacillus* to be described and is the first for which a genome sequence report is available. These data will provide a new perspective of how microorganisms adapt to anoxic and alkaline environments, and may also provide a pool of functional enzymes that work at higher pH.
